# Study protocol of DIVERGE, the first genetic epidemiological study of major depressive disorder in Pakistan

**DOI:** 10.1097/YPG.0000000000000333

**Published:** 2022-12-20

**Authors:** Maria Valkovskaya, Arsalan Hassan, Eirini Zartaloudi, Fahad Hussain, Muhammad Umar, Bakht Khizar, Inzemam Khattak, Shamshad Ahmed Gill, Shams-Ud-Din Ahmad Khan, Imtiaz Ahmad Dogar, Ali Burhan Mustafa, Moin Ahmed Ansari, Syed Qalb I Hyder, Muhammad Ali, Nilofar Ilyas, Parveen Channar, Nazish Mughal, Sumera Channa, Khalid Mufti, Ali Ahsan Mufti, Mian Iftikhar Hussain, Sadia Shafiq, Muhammad Tariq, Muhammad Kamran Khan, Shahzad Tahir Chaudhry, Abdul Rashid Choudhary, Mian Nizam Ali, Gohar Ali, Ashfaq Hussain, Muhammad Rehman, Noman Ahmad, Saeed Farooq, Farooq Naeem, Tanveer Nasr, Glyn Lewis, James A. Knowles, Muhammad Ayub, Karoline Kuchenbaecker

**Affiliations:** a Division of Psychiatry, University College London, London, UK; b Department of Pharmacy, University of Peshawar, Peshawar, Pakistan; c Institute of Clinical Trials and Methodology, University College London, London, UK; d Lahore Institute of Research and Development, Lahore; e The Brain Clinic & EEG Center, Sialkot; f Al-Shams Hospital, Sargodha; g Department of Psychiatry, District Headquarter Hospital, Faisalabad; h Department of Psychiatry and Behavioural Sciences, Sheikh Zayed Medical College/Hospital, Rahim Yar Khan; i Sir Cowasjee Jehangir Institute of Psychiatric and Behavioral Sciences, Hyderabad; j Ibadat Hospital; k Iftikhar Psychiatric Hospital, Peshawar; l Shafique Psychiatric Clinic, Peshawar; m Dr. Younas Khan Clinic, Charsadda; n SOLACE (Primary Psychiatric & Addiction Intervention Practice); o New Millat Brain Center, Sahiwal; p Swat Institute of Medical Sciences; q Department of Psychiatry, Saidu Teaching Hospital; r Swat Medical Complex, Swat; s Punjab Institute of Mental Health (PIMH), Lahore, Pakistan; t School of Medicine, Keele University, Keele; u Innovation Department, Midlands Partnership NHS Foundation Trust, Staffotdshire, UK; v Department of Psychiatry, University of Toronto; w Center for Addiction and Mental Health, Toronto, Ontario, Canada; x Human Genetics Institute of New Jersey (HGINJ), Rutgers University, New Brunswick, New Jersey, USA; y UCL Genetics Institute, University College London, London, UK

**Keywords:** depression, genome-wide association study, major depressive disorder, Pakistan, protocol, risk factor

## Abstract

Globally, 80% of the burdenof major depressive disorder (MDD) pertains to low- and middle-income countries. Research into genetic and environmental risk factors has the potential to uncover disease mechanisms that may contribute to better diagnosis and treatment of mental illness, yet has so far been largely limited to participants with European ancestry from high-income countries. The DIVERGE study was established to help overcome this gap and investigate genetic and environmental risk factors for MDD in Pakistan.

**Methods:**

DIVERGE aims to enrol 9000 cases and 4000 controls in hospitals across the country. Here, we provide the rationale for DIVERGE, describe the study protocol and characterise the sample using data from the first 500 cases. Exploratory data analysis is performed to describe demographics, socioeconomic status, environmental risk factors, family history of mental illness and psychopathology.

**Results and discussion:**

Many participants had severe depression with 74% of patients who experienced multiple depressive episodes. It was a common practice to seek help for mental health struggles from faith healers and religious leaders. Socioeconomic variables reflected the local context with a large proportion of women not having access to any education and the majority of participants reporting no savings.

**Conclusion:**

DIVERGE is a carefully designed case–control study of MDD in Pakistan that captures diverse risk factors. As the largest genetic study in Pakistan, DIVERGE helps address the severe underrepresentation of people from South Asian countries in genetic as well as psychiatric research.

## The DIVERGE study highlights

- Large sample size of South Asian participants
- Genome-wide genotyping for all participants
- Comprehensive assessment of diverse risk factors, incl. population-specific ones
- Detailed psychopathology assessment
- Focus on women’s mental health and hormonal factors
- Interview by trained mental health professionals
- Recontactable participants
- Recruitment at multiple sites across Pakistan for better representativeness
- Large proportion with severe Major Depressive Disorder
- Quasi matched controls to enable observational assessment of risk factors
- Co-led by local researchers
- Community engagement
- Data will be available via data access committee

## Introduction

Depression is a major contributor to the burden of disease affecting about 300 million people worldwide (Patel *et al*., 2016b; World Health Organization, 2021). Depression has a devastating impact on quality of life, high rates of comorbidity with physical illnesses and substantially contributes to suicide rates (Hawton *et al*., 2013; Patel *et al*., 2016a).

In low- and middle-income countries (LMICs), only an estimated 4% of affected individuals receive minimally adequate treatment (Thornicroft *et al*., 2017). Moreover, among patients with major depressive disorder (MDD) who get treatment, the majority experience relapse and about one-third recover only partially or remain chronically ill (Belsher and Costello, 1988; Mueller *et al*., 1999).

Furthermore, our understanding of the mechanisms underlying the development of MDD is crucial for the advancement of new biologically informed treatments. It is also essential for early MDD detection, which has been associated with better response and prognosis (Kraus *et al*., 2019). Furthermore, the existing classification systems for psychiatric illnesses lack biological validation, since they are predominantly based on self-reported symptom clusters and observable behaviours, instead of underlying neurobiological mechanisms (Clark *et al*., 2017). Research on the neurological and pathophysiological mechanisms, and the genetic architecture of MDD could help to identify biologically defined subgroups, hence providing additional evidence for the current classification systems, and reflecting neurobiological distinctions amongst clinical groups.

While genome-wide association studies have been successful in identifying genetic variants associated with MDD (Wray *et al*., 2018), the vast majority of participants were of European descent (Peterson *et al*., 2019). There are ongoing efforts using data from existing studies to increase the diversity of the genetic background of participants in studies investigating depression genetics (Giannakopoulou *et al*., 2021). However, due to the sparsity of data, individuals with South Asian ancestry are the most underrepresented ancestry group, and make up only 1.5% of the participants in the largest existing data resource. MDD is the result of a complex interplay of environmental and genetic risk factors (Kendler, 2012). Therefore, population-specific factors may play an important role, even in the context of genetic risk (Peterson *et al*., 2019; Giannakopoulou *et al*., 2021). Consequently, a better understanding of the cause of MDD requires dedicated studies in diverse global regions with genotyping as well as deep phenotyping.

The last decade has seen a strong trend towards big biobanks as well as low-cost recruitment, such as online questionnaires, to study common diseases. However, these approaches have several drawbacks. Rare and debilitating conditions are not well represented in these collections. Phenotyping for mental health has consistently been sparse, which is particularly problematic for such a complex condition as MDD (Cai *et al*., 2020) with heterogeneous cause and a large number of risk factors that interact with each other (Kendler, 2012). While being more limited in scope, carefully designed case–control studies are well-placed to consider the complexities of MDD.

### Depression in Pakistan

Pakistan is the sixth largest country in the world with a population that has increased drastically in the last few decades from 40 million in 1950 to 220 million to date (The World Bank, 2022a). There is extensive diversity in Pakistan with the Pakistani population divided into multiple ethnolinguistic groups and subgroups based on tribe/clan, profession and caste identity. The ethnolinguistic groups include large groups, such as Punjabis, Pathans, Sindhis and Balochis, and smaller groups, such as Muhajirs, Kashmiris and Hazaras. The divide is based on cultural and geographical differences, which contribute to varying living environments. Every major group has its own language, distinctive sociopolitical system features and a separate province/geographical area identified with their ethnicity. The Punjabis represent the largest ethnic group in Pakistan and have major representation in every sector of the country. This group is located on both sides of the India–Pakistan border. Pashtun ethnicity also comprises a considerable number and occupies the northwest of the country, along both sides of the border between Pakistan and Afghanistan. On the southeastern and southwestern sides of the country reside Sindhis and the Balochs, respectively (Hussain, 2005; Bhattacharya, 2015). Substantial parts of the population are exposed to risk factors for mental illness including dislocation, violence, political conflict, regional and political instability, and economic uncertainty (Mirza and Jenkins, 2004). There are tensions with regard to the current political situation and an immense number of recent refugees, in particular from neighbouring Afghanistan (Borthakur, 2017).

It is estimated that 50–70% of Pakistani marriages are endogamous, primarily between first and second cousins (Hussain and Bittles, 1998; Hina and Malik, 2015; Riaz *et al*., 2016; Arciero *et al*., 2021). The high rate of consanguineous marriages raises the possibility that part of the heritable component might work through recessive inheritance where a mutation is passed on from both parents (Martin *et al*., 2018). Hence, genetic studies of the Pakistani population provide opportunity for the discovery of recessive alleles and human knockouts of genes associated with traits and diseases. This together with high prevalence of psychosocial and socioeconomic stressors emphasises the potential of investigating the complex interplay between genetic and environmental risk factors for MDD in the Pakistani population.

Healthcare in Pakistan is provided by public and private sector facilities. For psychiatric disorders, however, there are no established referral pathways to specialist care, and the majority of service users have to pay out of pocket for the treatment. Publicly funded services are a preferred option for those who have limited capacity to pay (Naqvi *et al*., 2012).

Estimates of the prevalence of depression among adults in the community range from 25 to 60% for women and from 10 to 25.5% for men. The highest estimates were reported in questionnaire-based studies (Ali *et al*., 2002; Husain *et al*., 2007; Luni *et al*., 2009; Farooq *et al*., 2019) and studies with a two-stage design where participants were screened with a questionnaire and a proportion of high and low scorers was interviewed (Mumford *et al*., 1996, 1997, 2000; Husain *et al*., 2000). These high prevalence estimates may suggest that these studies measured psychological distress broadly.

The prevalence of MDD, when using more detailed assessments, was lower, such as 3.4% across both sexes (Nisar *et al*., 2004; Kausar *et al*., 2015). The estimates for women were higher (7.5%), with females three times more likely to be diagnosed than males (Nisar *et al*., 2004; Kausar *et al*., 2015). However, these estimates are likely to be affected by underreporting.

Among primary care patients, the prevalence of depression in rural and urban areas of Pakistan ranged from 23 to 60% (Dodani and Zuberi, 2000; Ayub *et al*., 2009; Athar *et al*., 2017; Ahmad and Hussain, 2018). The reported prevalence of MDD, according to a multisite interview-based study conducted in private and public sector primary care facilities in Lahore, was about 30% (Ayub *et al*., 2009).

In a general hospital among the patients referred for psychiatric consultation from other specialities, MDD was the most commonly reported diagnosis and accounted for 39% of all patients, with a majority being female (Yousafzai *et al*., 2015).

Depression: Interplay between Varying EnviRonments and GEnes (DIVERGE) is the first large comprehensive case–control study of MDD in Pakistan, and more widely in South Asia. Its aims are to investigate (a) the genetic influences of MDD, (b) nongenetic risk factors in the Pakistani population, and (c) their interactions.

DIVERGE is a part of the Pakistan Alliance on genetic RisK factors for Health (PARKH, https://www.genes-and-mental-illness.com) that also includes the GEN-SCRIP study about the GENetics of SChizophRenia in Pakistan and the GEN-BLIP study about the GENetics of BipoLar Disorder In Pakistan.

Here, we describe the DIVERGE study protocol: the interview we developed to capture diverse risk factors, including those of particular relevance to this population; the multi-site recruitment strategy to capture ethnic and regional diversity; and the genome-wide genotyping. We also describe our patient cohort using data from the first 500 patients. Finally, we discuss efforts to engage with the mental health patient communities in Pakistan.

## Methods

DIVERGE aims to enrol 9000 cases and 4000 controls for a clinical interview and a blood sample for DNA extraction. Recruitment occurs in multiple cities across Pakistan in both private and public clinics to capture ethnic diversity and ensure a wide representation of different socioeconomic strata (Supplementary Material, Supplemental Digital Content 1, http://links.lww.com/PG/A294). Cases are enrolled from Psychiatry Clinics or Outpatient Psychiatry Departments of Hospitals. This ensures the recruitment of moderate-to-severe MDD cases, which is likely to be advantageous for genetic discoveries. Referral is made by treating clinicians.

The study recruits participants aged 18 and above presenting with the capacity to provide informed consent. All Pakistani ethnic groups and those with regional migration background are eligible to participate. Individuals who meet the lifetime diagnostic criteria for any subtype of MDD, in accordance with the Diagnostic and Statistical Manual of Mental Health (DSM - editions IV, IV-TR, V) (American Psychiatric Association, 1994, 2000, 2013) and/or International Classification of Diseases (ICD-10, World Health Organization *et al*., 1992) criteria, are eligible to participate as cases. However, those with premorbid organic mental disorders, with a diagnosis of schizophrenia, bipolar disorder, or psychotic symptoms preceding depression onset, and with an MDD disease onset after 65 years old are excluded.

Controls are recruited from nonpsychiatry outpatient departments of the same hospitals or clinics. This strategy is implemented to recruit controls from the same population to enable comparisons of risk factors (Wacholder *et al*., 1992). Controls are proportionally matched to cases for their sex, age, socioeconomic status and ethnicity. Individuals without any personal history of MDD or previous suicide attempts are eligible to participate as control participants.

This protocol is a result of collaborative efforts between Pakistani and international researchers to cover expertise in psychiatry, psychology, medicine, genetic epidemiology, bioinformatics, and data science. The interview content was aligned with two large global depression studies, the Australian Genetics of Depression Study (Byrne *et al*., 2020) and the CONVERGE study of MDD in China (CONVERGE consortium, 2015), enabled through protocol sharing by the respective principle investigator.

**Fig. 1:**
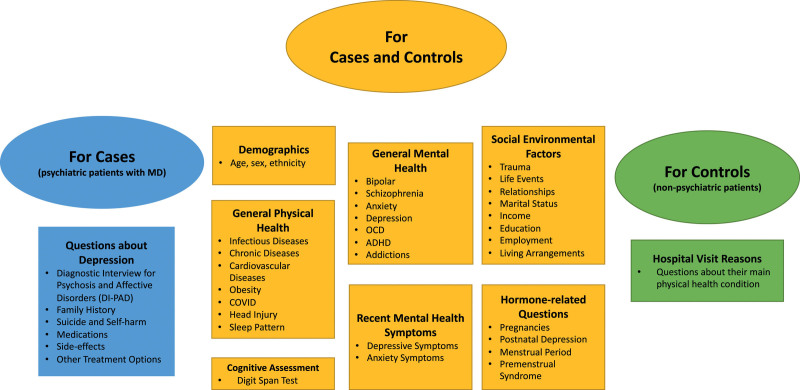
Overview of the content of the DIVERGE interview. DIVERGE, Depression: Interplay between Varying EnviRonments and GEnes.

The interview aims to capture potential risk factors for depression, including well-established as well as population-specific ones, and to provide an in-depth characterisation of the disease in the cohort. Questionnaires are administered through a structured interview to enable inclusion of participants with low levels of literacy and digital technology usage (Ameen and Gorman, 2009; The World Bank, 2022b). The interview lasts up to an hour and includes individual and household-related questions (Fig. 1 and Supplemental Material, Supplemental Digital Content 1, http://links.lww.com/PG/A294).

The interview questions were put together in English and translated into Urdu using a back-and-forth method in case there was no previous translation (e.g. COVID questions, medications section, suicide and self-harm, and hormone-related questions). Urdu is the official language in Pakistan and is widely spoken by the majority of the population (Aslam *et al*., 2022). Apart from Urdu, all interviewers speak a local language (Punjabi, Sindhi, Pashto, Balochi or Saraiki), common for the province where participants are recruited. If a question is unclear to a participant because of a language barrier, the interviewer provides an explanation in a local language. In some cases, the entire interview may be conducted in a local language. All interviewers underwent extensive training to avoid variations when interpreting questions in local languages.

### Mental health

All participants are screened for common mental health problems. Participants who report substance use undergo an addiction assessment with the Leeds Dependence Questionnaire (Raistrick *et al*., 1994). Nonpsychiatric participants recruited for the control arm presenting with depressive symptoms during the interview are not excluded and instead proceed to answer MDD-related questions. This group will be considered separately for most future analyses.

MDD patients are interviewed with the Diagnostic Interview for Psychosis and Affective Disorders (DI-PAD) (Pato *et al*., 2013). It includes questions about the lifetime symptomatology and the most severe MDD episode. In addition, suicidal behviour is assessed using an adapted version of the Suicide Behaviors Questionnaire-Revised (Osman *et al*., 2001), and a psychiatric medication history is extracted from prescriptions.

Hormonal changes in women may impact on risk of depression (Young and Korszun, 2010). The study, therefore, includes a set of hormone-related questions, for example, about pregnancy and menstrual periods (Kendler *et al*., 1992; Byrne *et al*., 2020). Postpartum depression is evaluated using the Edinburgh Postnatal Depression scale (Cox *et al*., 1987).

### Physical health

Health status is assessed by self-reports and covers a large number of common conditions. Additionally, control participants answer specific questions about symptoms and treatment related to the condition that led to their hospital referral.

### Environmental factors and socioeconomic questions

The interview also covers exposure to traumatic events as per the Life Events Checklist for DSM-5 (Weathers *et al*., 2013). Questions about displacement, migration, and refugee status have been added to the interview to reflect the local context.

Individual and household-related socioeconomic questions are included to reflect well-established risk factors and local practices, such as arranged marriage, early marriage, multiple spouses and living conditions. DIVERGE also covers a wide range of risk factors primarily related to women in Pakistan. These include sex inequality, a low level of educational attainment and intimate partner violence (Gulamani *et al*., 2013). Exposure to physical abuse is measured using the Women’s Experience with Battering scale (Smith *et al*., 1995), which we modified to open it up also to male participants. The Oslo Social Support Scale (OSSS-3, Kocalevent *et al*., 2018) is administered to assess social support, which can be a protective factor against depression.

### DNA collection

After the interview, on-site phlebotomists draw a blood sample. Blood samples are shipped to the UCL Genomics Centre in the UK for DNA extraction and genotyping using the Illumina Global Clinical Research Array with 1.2 million genetic variants. This microarray has been specifically developed to capture variation in global populations.

### Community engagement

Interviews will be carried out with a subset of participants to learn more about their views on the role of genetics for mental illness. Furthermore, a website https://www.genes-and-mental-illness.com/ with easy-to-understand information about the study has been created. Although scientific content about the genetics of mental health conditions is easily located online, little information is available about the genetics of mental illness aimed for the general public, in particular in Pakistan. Using engaging illustrations and translated in multiple languages, it aims to explain scientific information in lay terms. It also includes information about the genetics of other psychiatric and neurological disorders, incl. psychosis, eating disorders, dementia and intellectual disability.

The website includes a forum with an opportunity for community members to provide feedback on participating in the study and to share their views about research on genetics and mental illnesses. Although this way of community engagement might have limited applications for those participants with a low level of digital literacy, participants familiar with digital technology can benefit from accessible nonscientific content about the study and mental health in their own language.

## Results and discussion

### Demographics and socioeconomic variables

During the first month, the study recruited 500 participants (300 females) diagnosed with MDD in 10 data collection sites across Pakistan (Supplementary Table S1, Supplemental Digital Content 1, http://links.lww.com/PG/A294). All individuals provided informed consent, were confirmed eligible, and their data were included in the study for exploratory data analysis. The mean age of participants was 38 years old (SD = 10; range, 18–69) (Supplementary Fig. S1, Supplemental Digital Content 1, http://links.lww.com/PG/A294). Age was reported by all the participants.

**Fig. 2:**
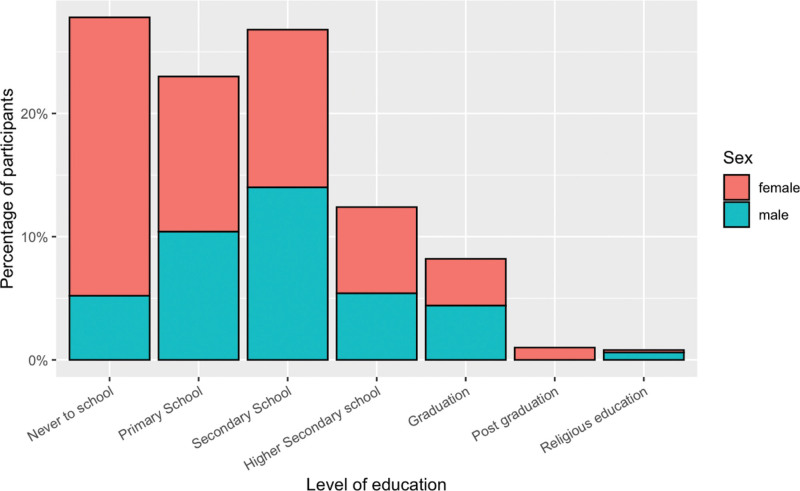
Level of education by sex for 500 participants with MDD. MDD, major depressive disorder.

Other demographic characteristics and socioeconomic variables captured in this study are presented in Table 1. The vast majority of participants were married (82%), and just over a half reported living in rural areas (55%). There was a substantial number who had not received any education (28%), with a higher proportion of females in this category (38% females vs. 13% males) (Fig. 2).

**Table 1:** Demographic characteristics of the DIVERGE cohort

Marital status	*n*	% (95% CI)
Married	412	82 (79–86)
Never married	45	9 (7–12)
Widowed	20	4 (3–6)
Divorced/separated	15	3 (2–5)
Engaged	8	2 (1–3)
Level of education		
Never to school	139	28 (24–32)
Primary school	115	23 (20–27)
Secondary school	196	39 (35–44)
Graduation	46	9 (7–12)
Religious education	4	1 (3–2)
Urbanicity		
Village	274	55 (50–59)
City	180	36 (32–40)
Town	46	9 (7–12)
Income		
No savings	326	65 (61–70)
Some savings	174	35 (31–40)

CI, confidence interval; *n*, number of participants.

### Psychopathology

**Fig. 3:**
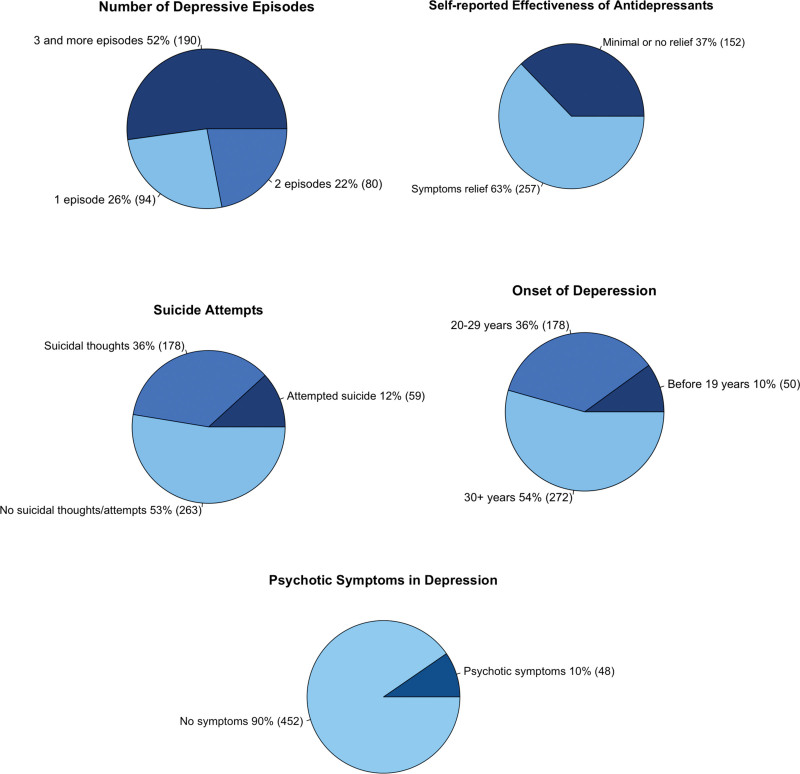
Clinical characteristics of the first 500 MDD patients recruited for DIVERGE. The pie charts reflect depression severity. The darker shades represent the severe form of symptoms. The percentage of participants and an actual count (in brackets) are reported for each variable. DIVERGE, Depression: Interplay between Varying EnviRonments and GEnes; MDD, major depressive disorder.

The analysis revealed that many participants in the cohort had severe depression (Fig. 3). The majority (74%) experienced multiple depressive episodes during the course of their illness. Almost half of the participants had the disease onset before 30 years old (Fig. 3 and Table S2, Supplemental Digital Content 1, http://links.lww.com/PG/A294), which is considered early depression onset (Byrne *et al*., 2020; Agerbo *et al*., 2021) and is associated with a more severe course of illness and other MDD risk factors, such as family history of psychiatric diseases and lower socioeconomic status (Liu *et al*., 2015; Wray *et al*., 2018; Agerbo *et al*., 2021). Over a third of participants reported the absence of or only minimal improvement of their depression from psychiatric medications. The prevalence of suicidal thoughts in our cohort (i.e. the number of participants who self-reported suicidal thoughts or attempts) was also 48% and is in line with the reported prevalence of suicidal ideations among severe cases, such as psychiatric inpatients with MDD (Cai *et al*., 2021).

Apart from depression, participants mentioned anxiety symptoms more often than other mental health problems (Table S3, Supplemental Digital Content 1, http://links.lww.com/PG/A294). Over a third of participants (37%) reported exposure to at least one traumatic event. This aligns well with reported prevalence of exposure to traumatic events in other LMICs (Dorrington *et al*., 2014). Among all traumatic events, natural disaster was the event most frequently reported by all the participants (17% of men and 15% of women). The rates of domestic violence were twice as high in women, compared with men, with 20% of females who reported aggressive behaviour from a close family member, although these rates tend to be underreported worldwide (Gracia, 2004; Babu and Kar, 2009; Huecker *et al*., 2022). Subsequently, 12% developed posttraumatic stress disorder symptoms; however, this number may vary between different regions in Pakistan (Sakuma *et al*., 2015) and will be reassessed in the full sample. In line with previous research (Gelaye *et al*., 2016), this study showed a high prevalence of both ante- and postpartum depression with approximately one in four women affected. One hundred sixty-nine patients reported a family history of mental illness among close relatives, the most commonly reported diagnosis being depression (34%) (Supplementary Fig. S2, Supplemental Digital Content 1, http://links.lww.com/PG/A294).

The majority (82%) reported a history of taking medications for their depression, whereas the rest received their first prescription at the time of referral to the interview.

Almost half (45% of all respondents) reported that they had received other forms of help for their depression, most frequently through a religious leader or a faith healer. Both terms are often used interchangeably and refer to traditional spiritual practice. This practice is widespread in Pakistan, and religious leaders and faith healers are usually the first points of contact for people who believe in the supernatural origin of mental illness and those who look for a religious cure, irrespective of their perception of the disease origin (Javed *et al*., 2020; Shafiq, 2020).

**Fig. 4:**
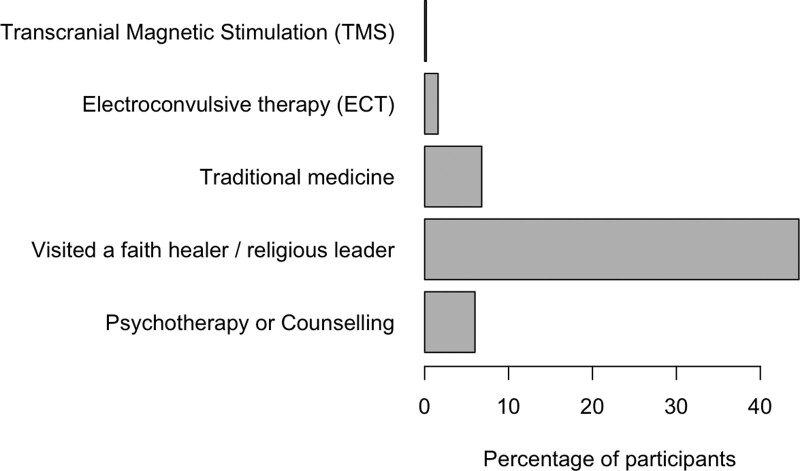
Forms of treatment and help for depression amongst 251 patients who reported any other help received for their current psychiatric diagnosis, apart from medications.

In line with other studies in LMICs (Cuijpers *et al*., 2018), only a small number of participants in this study (6%) had access to psychological therapies (Fig. 4).

### Limitations

Our study has some limitations. Patients are recruited in psychiatric hospital settings. This strategy restricts enrolment to those who have access to the healthcare system. Moreover, an observational case–control design is unable to establish temporal precedence of environmental factors with certainty and may be subject to biases, such as recall bias. Matching of controls in terms of hospital recruitment, demographic characteristics and ethnicity aims to reduce confounding when assessing risk factors. Another limitation of the study is that it restricted participation to those over 18 years old. This decision was made due to multiple reasons. First, because of the study’s cross-sectional design and inability to estimate the proportion of those whose MDD diagnosis would precede bipolar disorder onset (Beesdo *et al*., 2009). Second, for consistent usage of questionnaires to assess depression and its predominants in the cohort because a different set of tools is recommended for depression assessment in adolescents (Beirão *et al*., 2020). And third, due to concerns around the ability to enrol sufficient numbers of participants from younger age groups as access to specialist psychiatric care for adolescents in Pakistan is extremely limited (Khan *et al*., 2008). Finally, the reliability and validity of the socioeconomic questions and medication sections are unknown. The sections were developed specifically for this study in order to be able to capture population- and disease-specific factors. We do not intend to use them as scales.

### Conclusion

Recent years have seen a strong trend towards large biobanks, general cohorts and studies using electronic healthcare records. However, these offer very limited potential to investigate severe mental illness due to underrepresentation of patients or insufficient assessment of the complex risk factors and psychopathology. DIVERGE is a carefully designed case–control study of MDD in Pakistan. We described the study protocol that we developed to capture diverse risk factors of MDD, including those particularly relevant to the Pakistani population. We also presented a description of the first 500 patients demonstrating that most of the cases suffer from more severe forms of MDD. As the largest genetic study in Pakistan, DIVERGE will also help address the severe underrepresentation of people from South Asian countries in genetic research.

## Acknowledgements

The authors would like to thank all participants of this study, clinicians and administrative staff who made this research possible. The authors thank Professor Nick Martin, Professor Kenneth S. Kendler and Professor Jonathan Flint for kindly sharing their studies protocols. The authors would also like to thank Professor David B. Mumford for providing advice about measurement of depression symptoms.

DIVERGE is funded by the European Research Council (ERC) under the European Union’s Horizon 2020 research and innovation programme (Grant agreement No. 948561).

Conceptualisation: K. Kuchenbaecker and M. Ayub. Methodology: K. Kuchenbaecker, M. Ayub, A. Hassan, G. Lewis and J. Knowles. Data collection: A. Hassan, M.I. Hussain, K. Mufti, A.A. Mufti, M. Tariq, S. Shafiq, A.R. Choudhary, M.N. Ali, A. Hussain, G. Ali, M. Rehman, S.U. Din, A.B. Mustafa, I.A. Dogar, S.A. Gill, M.K. Khan, S.T. Chaudhry, S.Q. hyder, M. Ali, N. Ilyas, P. Channar, N. Mughal, S. Channa, M. Ansari, F. Hussain, I. Khattak, M. Umar, B. Khizar, T. Nasr, S. Farooq, F. Naeem and N. Ahmad. Data analysis: M. Valkovskaya and E. Zartaloudi. Writing - original draft preparation: M. Valkovskaya, E. Zartaloudi, A. Hassan, K. Kuchenbaecker and M. Ayub. Writing - review and editing: all authors. Funding acquisition: K. Kuchenbaecker. Supervision: K. Kuchenbaecker and M. Ayub.

Ethics approval: the study was performed in line with the principles of the Declaration of Helsinki. All procedures contributing to this work comply with the ethical standards of the relevant national and institutional committees on human experimentation. All procedures involving human subjects/patients, questionnaire and methodology were approved by the National Bioethics Committee of Pakistan (NBC-692). Ethical approvals have also been obtained from ethics boards of University College London (14125/002) and all collaborating institutes and hospitals where recruitment takes place.

Availability of data: the dataset analysed during the current study is available from the corresponding author on reasonable request. Upon completion of recruitment and genotyping, the DIVERGE data will be made available to bona fide researchers and access will be governed by a Data Access Co-ordinating Committee.

## Supplementary Material
