# Supplementary material for: Study protocol of DIVERGE, the first genetic epidemiological study of major depressive disorder in Pakistan
Source: Psychiatr Genet. 2022 Dec 20;33(2):69–78. doi: 10.1097/YPG.0000000000000333 (PMC9997631; doi:10.1097/YPG.0000000000000333)
Supplement: Supplementary file 1 [file pg-33-69-s001.pdf]

Study protocol of DIVERGE, the first genetic epidemiological study of major depressive disorder in Pakistan

Supplemental Digital Content

Content

Supplementary Text
- The Pakistani population structure
- Data collection sites
- Interview Details

Supplementary Tables
- Table S1 Age at recruitment of participants by sex
- Table S2 Age at depression onset
- Table S3 Participants reporting symptoms of other mental illnesses

Supplementary Figures
- Fig. S1 Age of participants by sex
- Fig. S2 Diagnosis of first- or second-degree relatives of 169 participants reporting a family history of mental illness.

Supplementary Text

The Pakistani population structure

There is extensive diversity in Pakistan with the population divided into multiple ethnolinguistic groups and subgroups based on tribe/clan, profession, and caste identity. The ethnolinguistic groups include large groups, such as Punjabis, Sindhis, Pathans, Balochis, and smaller groups - Muhajirs, Kashmiris, and Hazaras (Bhattacharya, 2015; Hussain, 2005).

Data collection sites

Participants are enrolled in multiple hospitals all over the country to capture the ethnic and socio-economic diversity of the Pakistani population. The enrollment is currently ongoing in the following areas: Charsadda, Faisalabad, Hyderabad, Islamabad, Karachi, Lahore, Peshawar, Quetta, Rahimyar Khan, Rawalpindi, Sahiwal, Sargodha, Sialkot, Swat.

Interview Details

Mental Health Introductory Assessment

All participants are asked questions about a range of mental health conditions, such as bipolar disorder, schizophrenia, depression, anxiety, panic attacks, OCD, and ADHD. Participants report if they experienced the main symptoms of any of these disorders, took relevant medications, and had ever received a formal diagnosis by a medical health practitioner. They are also asked about a family history of psychiatric illnesses. All participants answer questions about their alcohol consumption, smoking, and drug use. Those participants who answer *Yes* to alcohol or drug use undergo a more detailed assessment using the Leeds Dependence Questionnaire (Raistrick et al., 1994).

Physical Health and Neurological Conditions

Participants are asked *'Have you ever been diagnosed with any of these medical conditions or disorders'* and provided with a list of medical problems. Diseases are grouped into neurological and physical health conditions starting from those which represent exclusion criteria for this study (incl. dementia, epilepsy, and serious head injury). Physical health questions have been chosen based on the widespread comorbidity with depression (such as cardiovascular diseases) and common chronic health conditions in Pakistan (e.g. hypothyroid and diabetes). A range of other conditions has been presented to estimate the prevalence, such as COVID, cancer, vision problems, problems with digestion, and autoimmune disorders.

Depression Assessment

The Diagnostic Interview for Psychoses and Affective Disorders (DI-PAD) (Pato et al., 2013) is used to conduct a detailed assessment of depression according to DSM-IV and ICD-10 criteria. DI-PAD has a semi-structured design that allows interviewers (all experienced

clinicians) to elicit the most thorough information about the symptomology and pose additional probing questions if any information needs further verification. DI-PAD is specifically aimed to conduct a detailed assessment of psychiatric conditions based on interviewers' judgement. If uncertain, an interviewer has an option to include relevant information into designated note sections and make a judgement when the whole picture is captured and the interview is completed.

The DI-PAD section on depression includes questions about lifetime symptomatology and the most severe depressive episode. All interviewers undergo rigorous training in order to be able to administer DI-PAD. Although this tool is aimed to assess both Psychotic and Affective disorders, only sections relevant to depression assessment have been selected for this study as participants with schizophrenia and bipolar disorder were excluded based on the screener questions.

Suicidal behaviour assessment is based on The Suicide Behaviors Questionnaire-Revised (SBQ-R) (Osman et al., 2001). This concise instrument gathers information about lifetime suicidal behaviour and/or attempts and assesses the frequency of these during the past 12 months. Participants are also asked questions about self-harming behaviour.

<u>Medications and Other Treatment Options</u>

A section on medications is divided into three parts: psychotropics taken in the past, psychotropics that a participant has been taking up to visiting a psychiatrist on the day of the interview, and psychotropics prescribed at the current visit. This information is recorded from prescriptions that participants bring with them to the interview. Notes are taken about each prescription, including the dosage of medications. Participants are asked about side effects in relation to each prescription. Various side-effects are covered, incl. but not limited to dry mouth, sweating, nausea, vomiting, diarrhoea, headache, dizziness, fatigue, weight gain or loss. Participants have an option to mention a side-effect that is not on the list.

Participants are asked if they have received any help for their depression other than medications. The following answer options are included: "psychotherapy or counselling", "visited a faith healer / religious leader", " traditional medicine or other alternative treatment (e.g. Homoeopathy, Indian medicine)", "Electroconvulsive therapy (ECT)" and " Transcranial Magnetic Stimulation (TMS)". Participants are prompted to provide details of each treatment and how they think it helped their mental health. ECT questions are based on the Gen-ECT-ic data collection (Soda et al., 2020).

<u>Social environmental factors</u>

The interview includes socio-economic questions which reflect the cultural context. Participants are asked about their marital status, incl. polygamy marriage, as well as education, occupation and income, and living conditions. The Oslo Social Support scale is administered to find out about the social circle of family members and/or friends a participant can rely upon in difficult circumstances.

Life Events Checklist for DSM-5 (LEC-5) (Weathers et al., 2013) is used to learn about potentially traumatic events that participants might have experienced during their lifetime. A number of life events are read to participants. Participants can choose one or more answer options such as "Happened to me", "Witnessed it", "Learned about it happening to a close

family member or close friend", "Part of my job", "Not sure" and "Doesn't apply". Each event selected by a participant is followed by a question asking at what age did it happen and, in case it happened multiple times, what was the age of the first and the last time. Participants are also asked if the traumatic event has happened shortly before their depression onset. Some other potentially traumatic events not included in the original LEC-5 have been added, such as the death of parents or siblings, marital separation or parents, hospitalisation of close family members, financial issues, job loss, chronic illness, teenage pregnancy, divorce, and refugee status. Participants also answer questions about PTSD.

Hormone-Related Questions

Those participants who identify themselves as female are asked about pregnancies, postnatal depression, menstrual period, and premenstrual syndrome. These questions have been derived from the Australian genetics of depression study (Byrne et al., 2020) and combined with questions from a study about the Genetic and Environmental Factors in the aetiology of menstrual, premenstrual, and neurotic symptoms (Kendler et al., 1992). If applicable, participants report the number of pregnancies and live births deliveries, age of first menstrual period, menopause, heaviness and painfulness of the periods and method of contraception, and mood fluctuations before the menstrual period.

Cognitive Assessment

Working memory is tested by administering a digit span test. All participants listen to the same number of sequences starting from 2 digits and up to 8 digits. They are asked to repeat the sequences in the same order as read by an interviewer. A number of correctly repeated digits is noted down.

Supplementary Tables

Table S1 Age at recruitment of participants by sex

	sex	N (%)	mean	sd	median	range
**Age by sex**	males	200 (40%)	37.8	10.3	37	18-64
	females	300 (60%)	38.5	10.3	38	18-69

Table S2 Age at depression onset

	N	mean	sd	median	min	max	range
**Age at depression onset**	500	32.2	10.2	30	12	61	49

Table S3 Participants reporting symptoms of other mental illnesses

**Mental health symptoms**	**N of participants (%)**
Symptoms of anxiety (any type)	125 (25%)
Psychotic symptoms	48 (10%)
Marijuana/other drugs dependency symptoms	22 (4.4%)
Post-traumatic stress disorder symptoms	22 (4.2%)

ADHD symptoms	10 (2%)
Alcohol dependency symptoms	7 (1.4%)

Supplementary Figures

Fig. S1

Age of participants by sex

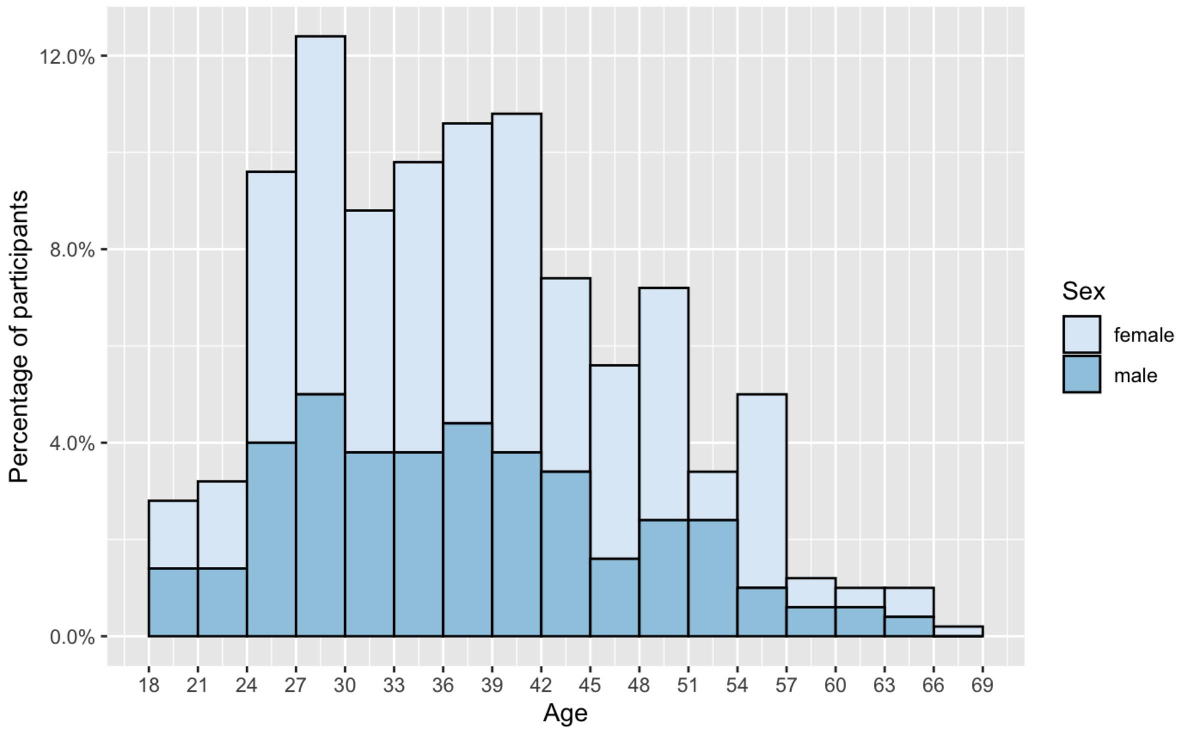

Fig. S2

Diagnosis of first- or second-degree relatives of 169 participants reporting a family history of mental illness.

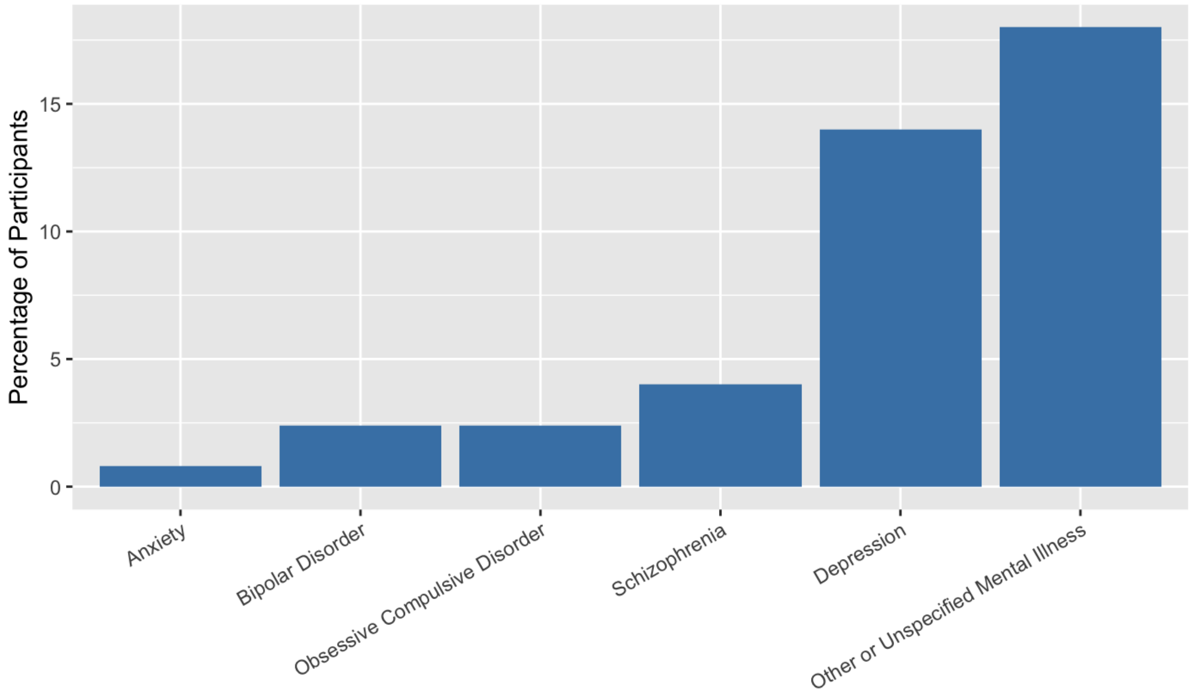

Note: **Other or Unspecified Mental Illness* includes predominantly those where the specific diagnosis was unclear as well as a small number of other psychiatric or neurological conditions (incl. intellectual disability, panic attacks, epilepsy).

References

Bhattacharya (2015) Pakistan’s ethnic entanglement. *The Journal of social, political, and economic studies*. academia.edu. Available at: https://www.academia.edu/download/38846981/JSPES_40_-_3_Bhattacharya_-_Pakistans_Ethnic_Entanglement.pdf.

Byrne EM, Kirk KM, Medland SE, et al. (2020) Cohort profile: the Australian genetics of depression study. *BMJ open* 10(5): e032580. DOI: 10.1136/bmjopen-2019-032580.

Hussain R (2005) The effect of religious, cultural and social identity on population genetic structure among Muslims in Pakistan. *Annals of human biology* 32(2). Taylor & Francis: 145–153. DOI: 10.1080/03014460500075167.

Kendler KS, Silberg JL, Neale MC, et al. (1992) Genetic and environmental factors in the aetiology of menstrual, premenstrual and neurotic symptoms: a population-based twin study. *Psychological medicine* 22(1): 85–100. DOI: 10.1017/s0033291700032761.

Osman A, Bagge CL, Gutierrez PM, et al. (2001) The Suicidal Behaviors Questionnaire-Revised (SBQ-R):Validation with Clinical and Nonclinical Samples. *Assessment*. DOI: 10.1177/107319110100800409.

Pato MT, Sobell JL, Medeiros H, et al. (2013) The genomic psychiatry cohort: partners in discovery. *American journal of medical genetics. Part B, Neuropsychiatric genetics: the official publication of the International Society of Psychiatric Genetics* 162B(4): 306–312. DOI: 10.1002/ajmg.b.32160.

Raistrick D, Bradshaw J, Tober G, et al. (1994) Development of the Leeds Dependence Questionnaire (LDQ): a questionnaire to measure alcohol and opiate dependence in the context of a treatment evaluation package. *Addiction* 89(5): 563–572. DOI: 10.1111/j.1360-0443.1994.tb03332.x.

Soda T, McLoughlin DM, Clark SR, et al. (2020) International Consortium on the Genetics of Electroconvulsive Therapy and Severe Depressive Disorders (Gen-ECT-ic). *European archives of psychiatry and clinical neuroscience* 270(7): 921–932. DOI: 10.1007/s00406-019-01087-w.

Weathers FW, Blake DD, Schnurr PP, et al. (2013) The life events checklist for DSM-5 (LEC-5). Instrument available from the National Center for PTSD.
